# Commentary for Special Issue on Using Baseline Target Moderation to Assess Variation in Prevention Impact: When (and How) to Revise Our Programs

**DOI:** 10.1007/s11121-022-01458-1

**Published:** 2022-11-23

**Authors:** Frances Gardner

**Affiliations:** Centre for Evidence-Based Intervention, Department of Social Policy & Intervention, University of Oxford, Oxford, UK

**Keywords:** Moderation, Mediation, Equity, Preventive intervention effects

## Abstract

Developing a better understanding of sources and mechanisms of heterogeneity is a key route to improving outcomes and targeting of preventive interventions. This commentary attempts to draw together findings from eight intervention trials in this special issue, each exploring baseline target moderation (BTM) or baseline target moderated mediation (BTMM). It considers their implications for prevention research and program design, particularly the question of whether they can help us to revise or adapt interventions. The studies cover a range of interventions, targets, and contexts, including parenting, couple, and CBT interventions, for depression, anxiety, conduct problems, or obesity. Some important findings stand out. Where studies found moderator effects, they tended to operate in a “compensatory” fashion, such that greater benefit was found in higher risk groups, suggesting that closer targeting might be warranted. It was rare for harmful effects to be detected for any subgroups. In other respects, patterns of BTM/BTMM findings were quite mixed across studies, suggesting it would be premature to change our interventions based on these trials. Implications of the findings for equity, for “slimming down” and scaling up interventions, and for research are discussed, including the need to combine BTMM with intervention component research, and to accumulate a more robust body of evidence by pooling data across trials.

## When (and How) to Revise Our Programs

The field of prevention science has been creative and successful in developing and rigorously testing many promising interventions. Nevertheless, intervention effects do not always replicate (Gorman, 2017), may be modest in size (Leijten et al., 2019), and often wane over time (Jeong et al., 2021), pointing to the need for further improving our science and interventions. A key avenue for improvement is to better understand sources and mechanisms of heterogeneity, through analysis of moderation and moderated mediation effects. By understanding which subgroups benefit most and least (baseline moderation analyses), we can affirm or improve the choices we make about targeting interventions, and can discern which subgroups may need altered, additional, or fewer interventions. Relatively few studies in the field however have taken this enquiry a step further, identifying those who respond differentially to intervention, and then exploring if there are distinct causal mechanisms in these subgroups (that is, moderated mediation analyses). This step is vital for generating hypotheses about how intervention components or delivery may be able to be “personalized” for different groups, in order to enhance effectiveness and reduce “waste,” where interventions have limited effects.

This special issue of *Prevention Science* brings together a body of work that has attempted to do just this with a focus on a theoretically important group of moderator variables. These are the baseline levels of the targeted behaviors or cognitions that the intervention (according to its theory of change) intends to influence (Howe, 2019). Generally, these are mediators along the causal pathway to change in the intended final outcome.

The eight studies in this special issue cover a range of interventions, targets, and contexts. Five concern parenting interventions, with a focus on preventing child depression (Brincks et al., this issue; Rojas et al., this issue), conduct problems (Weeland et al., this issue; Zhang et al., this issue), or obesity (Smith et al., this issue). These studies target hypothesized mediators of child outcomes, such as family functioning, or parenting skill, stress or emotion regulation. Other intervention types include a study (Helland et al., this issue) of CBT for childhood anxiety, targeting a child mediator, child emotion regulation. Two studies involve adult interventions: Myers et al. (this issue) evaluated an obesity prevention program targeting “well-being self-efficacy” as a mediator of change in subjective well-being; and Howe’s (this issue) aimed at preventing depression in couples facing unemployment, targeting multiple competing mediators, including couple communication, job search motivation, or sense of mastery. The findings are quite varied in terms of whether the authors’ hypotheses about baseline target moderated mediation (BTMM), or baseline target moderation (BTM), or mediation alone, were borne out by the findings. Consistency of findings was not high even across studies of interventions with similar theories of change. This commentary aims to bring together and examine the mixed patterns of BTM and BTMM results found in the papers, and consider their implications for research and for improving interventions.

## What Are the Implications of Finding BTM?

First, we look at studies that found hypothesized moderation by the variable that was targeted as an intermediate or ultimate outcome. Thus, Zhang et al. (this issue) found that military fathers with poorer emotion regulation benefited more from a parenting intervention in terms of it improving their emotion-related parenting practices, albeit only for one of four indices of the moderator variable. Rojas et al. (this issue) found that families with poorer family functioning benefited more from the Familias Unidas intervention, in terms of some youth outcomes. Howe (this issue) found that job seeking couples with lower levels of motivation and behavior around job searching, and males with higher depression, benefited more from the intervention. So far, all these moderators were found to operate in the “compensatory” direction, such that those in most need—at least in terms of the moderators tested—tended to improve most. However, many other tested moderators did not show effects, and a few went in the opposite, “rich get richer” — or even iatrogenic for some groups — direction, to that predicted. For example, Howe’s study (this issue) found a crossover effect, such that jobless couples with better communication skills benefited more, whilst a possible iatrogenic effect on depression was detected in couples with poorer communication at baseline.

We can draw some implications for intervention research and practice. Firstly, it is reassuring that in most cases where moderators are found, interventions are showing compensatory, rather than “rich get richer” effects. Very few harmful effects were detected. Secondly, interventionists might achieve stronger effects by targeting more closely those groups who benefit more, but it would be important to test a wider array of moderators, as it might be that other variables better serve to define who benefits most for a given outcome, as has been found in some studies of parenting interventions. For example, a large pooled data study found that children with highest baseline levels of conduct problems (the primary outcome and ultimate target of the intervention; Leijten et al., 2020), but not those with parents with poorer parenting skills (the key mediator; Gardner et al., 2017), benefited most. Targeting by level of parenting skill, despite its status in the theory of change, thus would not be the most helpful for defining groups who benefit most from parenting help.

Thirdly, where studies show potential harms for subgroups, then interventionists may need to consider how robust and plausible are the findings, and may need to step back into intervention development mode for this subgroup. This could take the form of gathering users’ views of the program elements and their possible harms, and developing and testing alternative components in pilot or microtrials. Howe suggests bolstering the couple communication component for the subgroup showing possible evidence of harm, but it is worth also considering if offering greater intensity of a harmful intervention is necessarily the best option, rather than focusing on alternative modes of intervention (for example, different models of couple communication, or individual interventions), which have not shown evidence suggestive of harm. Fourthly, these studies point the way to understanding differential mechanisms in subgroups — and these were tested in the BTMM analyses.

## What Are the Implications of Finding BTMM?

Next, we consider the implications of studies that, in addition to finding BTM, found some evidence of BTMM. This was the case for three studies (Zhang et al., this issue; Rojas et al., this issue; Myers et al., this issue), albeit generally the BTMM findings were not resoundingly clear-cut, applying only to some indicators of the moderator or outcome variables. As such, they offer partial support for the interventions’ theories of change — it is important, after all, for interventions at least to be able to show that key hypothesized mediating mechanisms work well in the subgroups that improve most. Some studies (Howe, this issue; Brincks et al., this issue) tested a series of (potentially additive or competing) mediating variables. This strategy holds promise for better understanding how to alter interventions by suggesting which mechanisms — and the intervention components associated with them — need to be emphasized, versus potentially downplayed for given subgroups. This is important for attempts to slim down and render more scalable our interventions. No studies showed evidence of different mediators working for different subgroups—if they did, this would provide convincing evidence for both the need to personalize interventions, by targeting different factors in different subgroups — and the potential means to do this. So far, the BTMM findings serve at best to affirm mechanisms for one group, but not yet to suggest what is needed — if anything — for other groups.

## What Are the Implications of Finding Mediation but no BTMM?

Two studies found predicted mediating mechanisms, but no moderation (Brincks et al., this issue; Helland et al., this issue). Thus, Helland et al. found that child emotion regulation mediated the effects of CBT on anxiety, but they did not detect variation in effects of CBT by initial level of emotion regulation. This pattern of findings suggests the interventions may be broadly applicable across different levels of the target, and given the proposed mediator seems to drive the outcomes, then we can be reasonably confident in the intervention working as intended in its theory of change. Further moderator and moderated mediation analyses might focus on whether there are other subgroups that benefit more or less, and if we can discern variations in the mechanisms, then that might indicate how we can help to bolster or alter intervention content or delivery for these groups. Equally, it might be that for some groups, mechanisms of change per se are not what need altering, but rather the cultural applicability of the approaches to change, or aspects of content or delivery, examples of which can be found in Parra-Cardona’s (this issue) commentary.

## Are We Ready to Revise or Adapt Our Interventions?

Considering the mixed picture of BTMM findings across studies, it appears we need to be cautious about changing interventions based on these data. Some of the studies show no BTMM or BTM effects, and others show them only for some predicted indices or constructs, and not others. For example, Zhang et al. (this issue) found BTMM in a parenting intervention for military fathers, but only for one of the four indices of emotion related parenting practices. Lack of effects may be due to low power, to measurement issues, or to interventions not operating via the mechanisms expected by their theory of change. Often BTMM analyses are conducted as secondary data analyses in a trial designed primarily to test main effects, meaning BTMM analyses may not have been preplanned or pre-registered, and the trial not suitably powered. If so, they should be treated as exploratory findings, and in need of replication. An important solution to low power is to pool data from multiple trials of similar interventions, known as Individual Participant Data (IPD) meta-analysis (Tierney et al., 2015), examples of which are seen in the family and parenting intervention studies by Weeland et al. and Rojas et al. (this issue), which have some of the largest sample sizes of studies in this special issue. Individual Participant Data meta-analysis brings further advantages, including enhancing our understanding of the robustness (or otherwise) of findings across contexts and populations, and bringing together collaborators across different trial teams to understand mechanisms (Perrino et al., 2013). Rojas et al. (this issue) is interesting in finding a different pattern of BTMM in a larger, pooled study, compared to the study by Brinck et al. (this issue) of the same intervention, Familias Unidas. This was despite considerable overlap in the participants in the two studies, with Brinck and colleagues’ trial contributing some 50% of sample for the pooled Rojas et al. data set. Differences in findings might be affected by the fact that Rojas et al. combined data from universal and indicated prevention trials, whereas the Brinck et al. sample was a universal one, meaning the baseline score ranges would likely be narrower. At the same time, this may give us pause for thought about robustness of findings — or could be viewed mainly as an argument for making greater efforts to enhance power and generalizability by pooling data across trials.

## Conclusions

Several pointers for the field can be drawn in conclusion. Firstly, the findings are quite variable in terms of whether BTM and BTMM hypotheses were borne out, and their consistency across indicators, meaning we might question their robustness. Secondly, few studies comment on the size and practical significance of any mediation and moderation effects found. Thirdly, replications and better powered studies are needed — in particular by pooling together the rich trial data we already have, using Individual Participant Data meta-analysis, before we are ready to change interventions.

Fourthly, an area showing more consistent findings was around the potential implications of BTM studies for equity. Where studies did show baseline target moderation effects, most suggested that equity is not likely to worsen (generally finding that “rich don’t get richer” — and in some cases, evidence of compensatory effects) as a result of employing these interventions. BTM analyses, however, only assess equity of outcome, not equity of access, and these may not work in the same direction. For example, one large pooled IPD study found no evidence that inequity of child *outcome* by ethnicity or poverty was likely to widen as a result of enrolling in a parenting intervention (Gardner et al., 2019). However, data the same set of trials found that *access* was patterned by level of poverty, despite many interventionists making strenuous efforts to engage low-income families (Berry et al., 2022). These data suggest the need to continue to check on equity effects of interventions, and to redouble our efforts to engage more marginalized families and communities.

What should we do when interventions do need to change? BTMM can suggest fruitful hypotheses about what to emphasize, drop, or change, and for whom, but mediators are not necessarily causal, and are not the same as intervention components. Personalizing interventions needs strong understanding of components, and mediators can help us to understand these. However, components need further testing before revising our interventions. There are many methods for doing this (Leijten et al., 2021), including analyzing what we already have, by using existing data in a components meta-analysis, as well as by directly testing new components in microtrials or factorial experiments (Collins et al., 2014).

What to do when we find indications of harms for subgroups? Should we bolster or remove components? Or should we revise our targeting to focus mainly on groups that benefit most? Or perhaps all of the above. We do, however, need to be cautious about whether our analyses were adequately powered to find these effects, and whether they are likely to be robust across studies and contexts. We also need to test new versions and components very carefully, in the ways suggested above. Shifting the way interventions are targeted may be a realistic and less costly adjustment to make, but depends on a careful assessment of many factors, based on the data and on community needs. Thus, would targeting the groups who benefit most mean that other needy groups miss out on interventions? Or, as suggested by many studies on moderators of parenting interventions for conduct problems (Leijten et al., 2019), would it serve to reduce unnecessary preventive efforts for those with lower need?

Finally, perhaps the trickiest and most important issue for prevention science to have public benefit involves how to take interventions to scale, whilst maintaining effectiveness and equity. BTMM, with its focus on the theoretically most important targets, in tandem with other methods for identifying essential components, holds much promise for helping decide what to emphasize in slimmer, better targeted interventions. BTMM can help select component combinations for developing slimmer versions of programs, which can be tested efficiently in factorial designs. Digital and hybrid interventions also hold promise for increasing scalability and reach. Scale-up also needs systems that support a workforce able to deliver and sustain affordable preventive interventions at scale (Shenderovich et al., 2021). These strategies are vital, if we are to develop interventions that — if effective — are more realistic to fund, disseminate, and sustain globally with quality and fidelity.
